# Hematology patients’ metaphorical perceptions of the disease and psychosocial support needs in the treatment process: a phenomenological study from a rural region of Türkiye

**DOI:** 10.1007/s00520-025-09278-z

**Published:** 2025-02-25

**Authors:** Sevda Uzun, Çiçek Ediz, Masoud Mohammadnezhad

**Affiliations:** 1 Department of Psychiatric Nursing, Faculty of Health Sciences, University of Gümüşhane, Gümüşhane, Türkiye; 2 Department of Psychiatric Nursing, Faculty of Health Sciences, University of Cukurova, Adana, Türkiye; 3 Faculty of Health, Education of Life Sciences, School of Nursing and Midwifery, Birmingham City University, Birmingham, UK

**Keywords:** Cancer care, Hematologic cancer, Metaphor analysis, Oncology nursing, Phenomenological study, Psychosocial support, Rural region

## Abstract

**Purpose:**

Although health services and access to these services have increased worldwide, there are still major barriers to access to health services, especially for rural, poor, and disabled individuals. The aim of this study was to evaluate the metaphorical perceptions of hematology patients living in rural areas in Türkiye about the disease and their psychosocial support needs during the treatment process with a phenomenological approach.

**Methods:**

In this study, in which the phenomenological research method was used, semi-structured in-depth interviews were conducted with 14 hematology patients receiving treatment in a state hospital in a province in the eastern region of Türkiye. Criterion sampling method, one of the purposive sampling methods, was used to reach the sample group. Interviews continued until data saturation was achieved. The data of the study were evaluated using thematic analysis. The study was conducted and reported according to the COREQ checklist.

**Results:**

Data analysis revealed two main themes including the following: metaphorical perceptions towards hematologic cancer with the sub-themes of emotional turmoil, social alienation and stigma and physical debilitation, and pathways to resilience with the sub-themes of disease process management, inner resilience and faith, and psychosocial support.

**Conclusion:**

This study revealed that the adaptation process of patients to hematologic cancer is quite difficult, and psychosocial support is an indispensable requirement for them in their lives. In order for patients and their families to cope with this very difficult disease process, it is thought that providing them with a higher level of psychological support will be beneficial in coping with the disease effectively.

**Supplementary Information:**

The online version contains supplementary material available at 10.1007/s00520-025-09278-z.

## Introduction

Hematologic disease is defined as a group of diseases caused by cells that can start in organs or tissues in any part of the body, grow uncontrollably, proliferate, and metastasize [1]. It is the second leading cause of death in the world [2] and in Türkiye [3]. According to the International Agency for Research on Cancer (IARC), the burden of cancer in the world is gradually increasing. For example, the number of cases of cancer increased from 18.1 million in 2018 to 19.3 million in 2020. In addition, the cancer death rate increased from 9.6 million in 2018 to 10.0 million in 2020. This situation shows that hematologic cancers are among the most common childhood cancers in the world, but they also constitute an important group of adult patients [1]. The incidence of hematologic cancers in Türkiye has been determined as 13.2 per 100.000 in women and 18.9 in men. Among hematologic cancers, non-Hodgkin lymphoma ranks first while leukemia ranks second in both men and women in Türkiye. The mortality rates of hematological cancers in Türkiye are in parallel with the WHO data with an estimation of 1 out of every 5 deaths [4].

Cancer is not only a physical disease but also a phenomenon that affects the individual psychologically [5]. From the stage of diagnosis to the terminal period, cancer profoundly affects the entire bio-psycho-social life of the individual. With the diagnosis of cancer, the presence of cancer diagnosis, the difficulty of cancer treatment, the side effects of treatment, the fear of the unknown, the fear of recurrence of the disease even if the disease regresses, and the fear of death change the whole life balance of the person and cause psychological distress [5, 6].

Cancer is an important disease that causes anxiety and depression, negatively affects the quality of life of patients, and therefore requires the determination of anxiety and depression levels of patients [7]. Hinz et al. (2010) found that the rate of anxiety and depression in cancer patients was 27.7% and 11.2% in the general population [8]. According to the results of the study, the prevalence of anxiety and depression disorders in hospitalized cancer patients is approximately twice as high as in the general population [8]. Some studies show that in addition to physical consequences, cancer patients have increased levels of anxiety and depression even years after diagnosis [9, 10]. It is stated that inadequate pain management in cancer patients causes an increase in anxiety levels, and the presence of high levels of anxiety increases pain and may cause sudden pain attacks in the patient [11, 12]. Accordingly, effective pain treatment and anxiety management in cancer patients are reported to be important in reducing symptom severity and improving quality of life [13]. Anxiety decreases the quality of life of patients and may reduce their compliance with treatment [14]. In addition to the diagnosis of cancer, treatment-related symptoms also increase the anxiety of the individual with cancer. Considering all these reasons, the process experienced by cancer patients due to the disease itself, the toxicity and symptoms caused by the treatment should be evaluated and their anxiety should be defined [15].

Continuing the treatment in parallel with effective care and providing adequate social support to the individual facilitates the patient’s adaptation to the disease and makes the treatment process easier to overcome psychologically and physically. In this process, the needs perceived by patients and the service provided must be in harmony. Incompatibility between care and needs leads to unmet needs. Meeting the needs reduces the patient’s anxiety and dissatisfaction with care and improves the quality of life [16]. In a study by Morrison et al. (2012) investigating unmet needs in cancer patients, it was shown that information and social support were the most needed needs [17]. Although health services and access to these services have increased worldwide, there are still major barriers to access to health services, especially for rural, poor, and disabled individuals. Since individuals live in rural areas, they face significant problems in reaching the city and applying to health institutions. In addition to transportation barriers, language, culture, and poverty also make it difficult for individuals to use health services adequately. Especially in rural settlements in Türkiye, the inadequacy of professional psychosocial support systems has a negative psychosocial impact on individuals with cancer. All these negative factors affect how individuals perceive their illness and what their psychosocial needs are [18, 19]. In this context, this study aimed to evaluate the metaphorical perceptions of hematology patients living in rural areas in Türkiye about the disease and their psychosocial support needs in the treatment process with a phenomenological approach.

## Methods

Throughout this study, the authors followed the Consolidated Criteria for Reporting Qualitative Research (COREQ) [20] and reported accordingly (Appendix 1).

### Study design and setting

This qualitative study was conducted between July and August 2024 using a descriptive phenomenological design. A phenomenological design aims to understand a phenomenon by exploring it from the perspective of people who have experienced it [21].

This phenomenological design is suitable for exploring hematology patients’ metaphorical perceptions of their disease, and their psychosocial support needs during treatment.

The study was conducted in the Hematology Outpatient Clinic of Van Health Sciences University Van Training and Research Hospital. In determining the hospital where the research will be conducted, the fact that Van province is one of the easternmost provinces of Türkiye, the province with the highest population in the eastern region [22], the accessibility of the researcher and the cooperation of the team with the researcher were taken into consideration.

### Study participants

The criterion sampling method, which is one of the purposeful sampling methods, was used to determine the study group of the research. Criterion sampling is the sampling of people, events, objects, or situations that have the qualities determined for the problem [23, 24]. The inclusion criteria were as follows: (a) being diagnosed with hematologic cancer and being in any stage of the disease, (b) being open to communication, (c) residing in a rural area. The exclusion criteria were as follows: (a) having speech, language, or hearing impairments.

### Data collection tool

A semi-structured open-ended interview guide was used to collect data. The interview guide was prepared by the researchers based on the literature review and in line with the research aim. In the interviews, the patients were asked to describe their thoughts about the situations experienced by hematology patients. The interview consists of two parts. The first part includes questions such as age, gender, diagnosis, and how many years the individual has been a hematology patient. The second part included six basic open-ended questions in order to ensure the content and face validity of the interview guide, the opinions of 3 faculty members who are experts in the field of mental health nursing were obtained. Based on the expert opinions, the interview guide was finalized by making additions and corrections (Table 1). The interview guide was prepared in Turkish, and all interviews with the patients were conducted in Turkish (Table 1).

**Table 1:** Questions in the semi-structured interview form

1	What does the word cancer mean to you? What does it mean to you? How did you feel when you were diagnosed? What emotions did you experience? (Cancer is like….. because was asked to complete the metaphor.) (After the participant answered; “Is it curable? What are the effects of cancer on you?)
2	With whom and how did you first share this situation? What kind of reactions did you receive from your spouse and/or relatives? How did you feel in the face of these reactions?
3	Do you share with others that you are a hematology patient (after the participant answers; What are the reasons?)
4	How did you manage the process? What are the situations that contributed positively or negatively to your successful or unsuccessful management of the process? What do you think about your sources of support in this process? Do you think you are in the process of adaptation? What are the reasons?
5	What are the needs of hematology patients in the community and health centers? What would you like to have as social support?
6	If individuals with hematologic diseases were to be educated, what would you want to be explained? In which subjects do you think hematologic patients need information?

A pilot study was conducted with three patients diagnosed with hematology in order to improve the comprehensibility and applicability of the data collection forms and the ability to obtain data appropriate to the research questions and to improve and standardize the interview skills of the researcher. As a result of the pilot study, there was no need to make any changes to the questions. However, due to the standardization of the interview, the patients included in the pilot study were excluded from the main study.

### Study procedure

All potential participants were informed about the purpose and procedure of the study through announcements by the hematology physician contacted by the second investigator 1 week before the interviews began. Those who met the study criteria were asked to read an information sheet and sign a consent form. The Ç.E. conducted all interviews after scheduling face-to-face interviews that were feasible for both the interviewer and the interviewee in private rooms in the outpatient department of the hospital. Each interview was conducted in Turkish, lasted 30–45 min, and was recorded.

### Data analysis

All interviews were transcribed Ç.E., and triangulation was checked by the other researchers. Thematic analysis was used to analyze the data. To do this, each transcript was read and re-read by the second researcher and the other researchers. Thus, the most frequently repeated sentences were found in each interview. Codes were identified using a codebook, and sub-themes were identified by combining similar codes; common themes were identified by combining similar sub-themes [25]. If the codes were not similar, a discussion was held between the two coders to resolve the difference. The discussions continued until a joint decision was made and ended when a joint decision was made.

### Study rigor

In a qualitative study, it is important to ensure rigor and reliability. In this study, reliability is based on four measures: credibility, dependency, verifiability, and transferability [26, 27]. For credibility, all participants were informed about the purpose and study procedure verbally and using an information sheet before inclusion in the study. All participants were asked to sign a consent form. Interviews were conducted at a time and place convenient for both the interviewer and the interviewees. The second researcher had basic knowledge about the conduct of the interview and data collection. The interview instrument was piloted before it was applied to the interview. All interviews were recorded with a digital recorder and transcribed on the day of the interview. For reliability, the transcriptions were re-read to correct all possible errors. Two independent coders read all transcripts, separately identified codes, and discussed differences. For verifiability, all researchers discussed and validated the identified codes, themes, and sub-themes. Transcription was done using purposive sampling and continuing the interviews until data saturation was reached.

Permission to conduct the interview was obtained from the responsible physician in the field of hematology at the selected public hospital. All participants were informed about the purpose and objectives of the study, possible benefits or harms, and their right to participate in the study using an information form. Those who met the study criteria were asked to sign the consent form. The information sheet and consent form were provided to the participants in Turkish. They were informed that participation in the study was completely voluntary and that they could terminate their participation at any stage. Participants were kept anonymous, and all information was kept confidential [28, 29].

### Research team and reflexivity

All three members of the research team are active faculty members (associate professors) in nursing schools. Two of the researchers have a doctorate in psychiatric nursing and one in public health nursing. Two of the researchers have experience working as clinical nurses in hospitals and have received training in qualitative research methods. All three researchers have experience in qualitative interviewing and have qualitative studies in international journals.

### Ethical considerations

This research was approved by the X University Scientific Research and Publication Ethics Committee (dated 24.08.2023, numbered 2023/96:1) Written informed consent was obtained from the participants before starting the interview. Verbal informed consent was also obtained again at the beginning of the interview. Recordings and transcripts were stored on a password-protected device. The study was conducted in accordance with the Declaration of Helsinki and the ethical standards of the National Research Committee.

## Results

The mean age of the individuals included in the study was 48.50 ± 14.34, and the mean duration of illness was 4.5 ± 1.22. The participants were between 24 and 66 years old and eight of them were female. In addition, six of the participants were primary school graduates (Table 2).

**Table 2:** Characteristics of participants

Participant number	Age	Gender	Marital status	Education status	Diagnosis	How many years of hematologic cancer diagnosis	At what stage of the disease
P1	52	Male	Married	University	Chronic lymphocytc leukemia	7 years	In the last phase of treatment
P2	58	Female	Single	High school	Chronic lymphocytc leukemia	4 years	Follow-up phase
P3	66	Female	Single	Literate	Marrow cancer	3 years	Follow-up phase
P4	61	Male	Married	Primary school	Leukemia	5 years	Treatment phase
P5	58	Male	Married	Primary school	Acute lymphocytic leukemia	3 years	Harmonization process
P6	29	Female	Single	High school	Hodgin lymphoma	4 years	Follow-up and control phase
P7	24	Male	Single	Primary school	Hodgin lymphoma	4 years	Follow-up and control phase
P8	69	Male	Married	High school	Lympho leukemia	4 years	Follow-up and control phase
P9	34	Male	Married	High school	Hodgin lymphoma	5 years	Treatment phase
P10	45	Female	Married	High school	Myleoid leukemia	3 years	Follow-up and control phase
P11	31	Male	Single	High school	Hodgin lymphoma	4 years	Treatment phase
P12	44	Female	Single	Primary school	Chronic lymphocytc leukemia	5 years	Treatment phase
P13	56	Male	Married	Primary school	Hodgin lymphoma	6 years	Treatment phase
P14	52	Female	Single	Primary school	Hodgin lymphoma	6 years	Treatment phase

Themes, sub-themes, and codes were identified (Table 3).

**Table 3:** Summary of themes, sub-themes, and open codes identified

Themes	Sub-themes	Codes
**Metaphorical perceptions towards hematologic cancer**	**Emotional turmoil**	Shock, pessimism, hopelessness, denial, acceptance, frightening situation, grief, mourning, despair, mental breakdown, sadness, loss, death, grief, confusion
	**Social alienation and stigma**	A disease that cannot be shared with the community, social isolation, stigma due to deterioration in body image, a difficult journey
	**Physical debilitation**	Fatigue, chronic pain, fatigue, weakness, loss of appetite/weight loss, hair loss
**Pathways to resilience**	**Disease process management**	Accepting the situation, getting over the shock phase quickly, starting treatment, not being able to share the illness with anyone outside the family, getting family support, facing the situation
	**Inner resilience and faith**	Self-acceptance, praying, taking refuge in spirituality, dreaming, getting psychological support, spending time with family, getting social support, getting away from society
	**Psychosocial support**	Providing information about the disease, changing the society’s perspective on cancer, providing treatment and follow-up priority to hematology patients in health institutions, providing economic support, providing psychological support, creating special areas in hospitals for hematology patients, providing training on drug side effects and things to pay attention to, providing transportation support for patients living in rural areas, creating a mobile application for hematology patients

### Theme 1. Metaphorical perceptions towards hematologic cancer

This theme includes three sub-themes regarding patients’ metaphorical perceptions of cancer as explained below. These sub-themes are emotional turmoil, social alienation and stigma, and physical debilitation.

#### Subtheme 1. Emotional turmoil

According to the data obtained from the interviews, it was determined that individuals’ metaphorical perceptions of cancer included situations such as fear, pessimism, hopelessness, helplessness, and mental collapse.

It has the connotation of a bad disease. It is very difficult to treat if it is too late, but it is a treatable disease with early diagnosis. At the beginning, I had extreme fatigue and restricted movement. After I started medication, the fatigue I felt gradually decreased. Psychologically, I had a serious psychological breakdown at first, but after I recognized the disease, I started to recover because I believed that I could beat this disease (P1, Male).

It has been determined that cancer means mourning and death for some individuals and that they are negatively affected by the losses they experience due to cancer.

The word cancer evokes very bad feelings for me. It means loss, mourning, death for me. When I first learned the diagnosis, I could not accept it for a long time, I could not believe it, I cried a lot. But then I accepted it (P3, Female).

#### Subtheme 2. Social alienation and stigma

According to the data obtained from the interviews with the participants, individuals associated cancer with a disease that cannot be shared with the society, social isolation, and experiencing stigma due to deterioration in body image.

I learned about my illness by chance that day when I applied to the hospital with my wife and daughter with complaints of high blood pressure. Although my wife and daughter are health professionals, they were also shocked at first. I shared my illness only with my nuclear family. They supported me and said that we would overcome the treatment process together. I did not want to share it with anyone other than my nuclear family. I didn’t want them to look at me as if I had a bad disease and evaluate me like that because it would affect me psychologically worse, so I didn’t want to share it with others (P1, Male).

#### Subtheme 3. Physical debilitation

Interviews with the patients revealed that participants’ metaphorical perceptions of cancer included hair loss, sleep problems, fatigue, weakness, and loss of appetite.

When I first heard the word cancer, I thought of my hair. For me, cancer meant that my hair would fall out. I was very scared when I first learned about it. I never thought it could happen to me. The first thing I was worried about was how to share it with my environment. I experienced the stress of what kind of reaction I would face, what they would ask me and how I would answer their questions (P6, Female).

Another participant stated that:

At first I felt very bad, but I tried not to lose faith in my recovery. Physically I lost a lot of weight, I felt weak and very tired (P5, Male).

### Theme 2. Pathways to resilience

This theme includes three sub-themes: disease process management, coping and psychosocial support, as explained below.

#### Subtheme 1. Disease process management

Participants stated that they had great difficulty in managing the disease process in hematologic cancer.

Honestly, this process was up and down. Sometimes I had physical pain, I was worried at those moments. I would immediately call my doctor, he would support me and I would feel better. There were moments when I felt hopeless from time to time, but there were also moments when I felt good. In this disease, the support of my relatives and my doctor affected me a lot in a positive way (P7, Male).

#### Subtheme 2. Inner resilience and faith

Participants reported that they tried to cope with the disease process by using situations such as self-acceptance, prayer, spirituality, and daydreaming.

When I first received the diagnosis, I didn’t know how to manage it. I had mixed emotions, but I tried to manage it by listening to my doctor and my family without losing my morale. My chemotherapy process lasted four months. During this time, I had moments of psychological decline, but there were also moments when I felt strong. But as a result, I think I successfully overcame the chemotherapy process. My biggest source of support was my family. With the strength they gave me, my belief that I would get through it increased. (P6, Female)

Another participants stated that:

At first, I floundered a bit because I could not accept it for a long time. I could not manage the first process successfully because I did not have a family to support me. But then I decided to get treatment with the support of my sister and close friends with whom I shared the situation. With their support, I managed the process more successfully. I can say that I am now in a complete adaptation process (P2, Female).

#### Subtheme 3. Psychosocial support

Individuals stated that there should be a separate follow-up center for them for the disease process, so that their treatment would be more effective and they would feel better mentally.

Hospitals should have a separate follow-up center to explain the whole process to hematology patients and follow them. They should deal with patients not only with their medical treatment but also with their mental state. In this process, we may experience psychological decline from time to time, which is reflected in the response we will receive from medical treatment. Therefore, hematology patients should be handled and supported in all aspects. In fact, I think there should be a mobile line that we can always reach and share the problems we experience and ask questions (P8, Male).

In addition, patients stated that providing them with education on the side effects of medication and things to be considered would provide important psychosocial support for them.

I had a lot of questions after I received this diagnosis, but I didn’t have a doctor who could adequately explain any of them. They only gave me information about the medication I would be taking for treatment, but they didn’t give me any holistic information about what I should pay attention to in general from now on. For example, I was wondering how I should eat, or what I should pay attention to in my daily activities. They don’t give general information. There is also a psychological dimension to this process. But doctors do not look at it from this perspective. We were told that our mood is very important in the treatment. But the health center did not provide any support for this (P9, Male).

## Discussion

In this study, it was aimed to evaluate the metaphorical perceptions of hematology patient living in rural areas in Türkiye about the disease and their psychosocial support needs in the treatment process with a phenomenological approach. The discussion was addressed in two themes: metaphorical perceptions towards cancer and pathways to resilience.

### Metaphorical perceptions towards hematologic cancer

It was determined that the metaphorical perceptions of hematology patients towards cancer were generally negative themes. When the studies were examined, it was determined that many patients used both journey and war metaphors to make sense of their cancer diagnosis or treatment [30, 31]. In journey metaphors, patients may have a “challenging road” ahead of them and reach a “crossroads” or “progress” with treatment. In war metaphors, patients have an “enemy” or “struggle” with their illness and become a “survivor” [32]. In cancer patients, fear due to the known or unknown characteristics of the disease, the thought that the treatment will not work, thinking that they will get negative results, not receiving enough support from the family while hospitalized, experiencing uncertainty, infections, metabolic disorders, brain metastases, and especially the treatments (chemotherapy, radiotherapy, steroids, antiemetics, etc.) can lead to anxiety [33].

In line with the results of the study by Johansson et al. (2012), participants with allogeneic stem cell transplantation identified the importance of regaining mental energy and being able to cope with anxiety and uncertainty about the disease and treatment and tried to understand what was happening to them [34]. Dunn et al. (2016) reported that they experienced a major disruption in their lives physically, psychosocially, and emotionally, including coming to terms with their own mortality, without a sense of when they would be able to return to normal [35]. Cancer is a chronic disease that affects the lives of individuals and their families due to its prognosis and treatment method. In many studies in the literature, it has been found that individuals diagnosed with hematologic cancer are affected by quality of life and experience psychological and social problems [36]. Although the results of the study are in parallel with the literature, it can be said that cancer affects individuals in all aspects.

### Pathways to resilience

It was found that patients had great difficulty in disease process management and had problems in coping. It is reported that social support has a significant effect in crisis situations and affects the mental and physical health outcomes of individuals. It is stated to be a source of support that facilitates psychological adaptation, especially in diseases such as cancer [33]. During the diagnosis and treatment of cancer, families are expected to provide emotional, financial, and informational support to the patient to increase patient compliance. Although cancer is seen as a problem that concerns the whole family, there are few studies examining the effect of family relationships on patients’ adherence to the disease. On the other hand, patients reported more chemotherapy-related physical symptoms when their families lacked such characteristics [11]. Although the characteristics of family relationships are generally seen as stable, a major stressor such as the occurrence of cancer in a family member can change family relationships [14]. Therefore, social support is thought to be one of the issues that should be supported with more studies. The process of managing the side effects of cancer during and after treatment requires a multidisciplinary approach. Individuals are affected both physiologically and psychologically in accepting the disease, in the state of uncertainty caused by the process, and in coping with the side effects of the treatment, especially during the diagnosis and treatment processes. They need family support the most in managing this stage [14, 35]. Patients who do not receive adequate social support have more difficulty in coping with this already difficult process. Studies have shown that there is a positive relationship between lack of social support and anxiety levels in cancer patients. In patients who do not receive adequate support during the disease process, feelings such as loneliness, feeling incomprehensible, and inadequacy can increase anxiety levels; on the other hand, it can also occur because previous experience of nausea and inability to manage it appropriately cause fear of reliving this symptom [14]. In line with the results of the study, it can be said that psychosocial support is a very important condition for individuals to cope with the disease.

## Limitations

One of the limitations of the study is that all participants were selected from a province in the eastern region of Türkiye. The results depend on the participants and the setting in which the study was conducted. The participant group is not representative of the entire population of individuals with hematology cancer.

### Implications for nursing policy

Hematologic cancer is a complex health issue with biopsychosocial dimensions that can inflict deep wounds on both the individual and their immediate environment. Although nurses evaluate individual patients with hematologic cancer from a biopsychosocial perspective while fulfilling their responsibilities, they should not forget the impact of their beliefs, attitudes, and behaviors regarding cancer on individuals with hematologic cancer. Nurses can use effective psychosocial interventions to impart knowledge related to cancer management, organize group therapy (i.e., peer support), perform to ensure continuity of treatment, and improve individuals’ self-management skills. In this process, initiatives such as stress management, interpersonal relationship, and problem-solving skill improvement for the individual and family should be planned and implemented.

## Conclusions and recommendations

Patients with hematologic malignancies experience an existential crisis, severe uncertainty, and significant biopsychosocial problems throughout their illness. This study revealed that the process of adaptation to the disease is very difficult, and psychosocial support is very important for individuals in their lives. It is thought that providing higher levels of psychological and social support to patients and their families to cope with this difficult disease process will be beneficial for individuals to cope effectively with the disease. On the other hand, it was concluded that individuals experienced significant problems in disease process management, both in accessing health services and in treatment areas in health centers, and that these problems negatively affected the treatment process*.* In addition, challenges specific to rural areas such as lack of mental health services and cultural stigmatization also negatively affected the way individuals coped with the disease.

To address hematologic patients’ issues, the government should support and prioritize treatment and follow-up of hematology patients who are living in rural areas. In addition to economic and care support, psychological support should also be provided for the mental health of patients living alone. Patients should easily access comprehensive information about the disease and the treatment process using digital devices and mobile applications. In order to change society’s perspective on cancer, it is recommended to develop awareness programs at the community level, to draw attention to the importance of early diagnosis, to ensure that people undergo screenings, to establish awareness-raising centers within hospitals, and to work effectively.

## Supplementary Information

Below is the link to the electronic supplementary material.

Supplementary file1 (DOCX 18 KB)

## Data Availability

Data is provided within the manuscript or supplementary information files.
