# Supplementary material for: Hematology patients’ metaphorical perceptions of the disease and psychosocial support needs in the treatment process: a phenomenological study from a rural region of Türkiye
Source: Support Care Cancer. 2025 Feb 25;33(3):222. doi: 10.1007/s00520-025-09278-z (PMC11860990; doi:10.1007/s00520-025-09278-z)
Supplement: Supplementary file 1 — Supplementary file1 (DOCX 18 KB) [file 520_2025_9278_MOESM1_ESM.docx]

**Appendix 1. Combined criteria for reporting qualitative research (COREQ).**

**Area 1: Research team and reflexivity**

**Personal Characteristics**

| **Number** | **Item** | **Guiding questions** | **Explanations** |
| --- | --- | --- | --- |
| **1** | Interviewer/facilitator | Which author(s) conducted the interview or focus group? | The second author conducted the interview**.** |
| **2** | Credentials | What were the credentials of the researcher, e.g. PhD, MD | First author PhD  Second author PhD  Third author PhD |
| **3** | Profession | What was their occupation at the time of the study? | First author Associate Professor Psychiatric Nursing  Second author Associate Professor Psychiatric Nursing  Third author Associate Professor Public Health Nursing |
| **4** | Gender | Was the researcher a man or a woman? | Two researchers Female  A researcher Male |
| **5** | Experience and training | What experience or training did the researcher have? | All three authors have taken qualitative courses, have experience in qualitative research, and have published qualitative studies in international journals. |

**Relationship with participants**

| 6 | Relationship status | Was a relationship established before the training started? | No relationship was established before the start of the study. |
| --- | --- | --- | --- |
| 7 | Interviewer's participant knowledge | What did participants know about the researcher, e.g. personal goals, reasons for doing research? | Participants knew that the researcher had a PhD in mental health and diseases and public health nursing |
| 8 | Interviewer characteristics | What characteristics were reported about the interviewer/facilitator, e.g. bias, assumptions, motives and interests in the research? | At the beginning of each interview, individuals were informed about the purpose and objectives of the study. |

**Area 2. Study design**

**Theoretical framework**

| 9 | Methodological orientation and Theory | Which methodological orientation was specified to support the study, e.g. discourse analysis, ethnography, phenomenology, content analysis? | This was a phenomenological study. |
| --- | --- | --- | --- |

**Participant selection**

| 10 | Sampling | How were participants selected? e.g., purposeful, convenience, consecutive, snowball | A purposive sampling method was used. |
| --- | --- | --- | --- |
| 11 | Approach method | How were participants approached, e.g. face-to-face, telephone, mail | The timing of the interviews was determined by the individuals who voluntarily agreed to participate in the study. |
| 12 | Sample size | How many participants were there in the study? | A total of 18 individuals were included in the study. |
| 13 | Disagree | How many people refused to participate or dropped out? Reasons? | There were no individuals who refused to participate in the study. |

**Setting**

| 14 | The setting of data collection | Where was the data collected? e.g. home, clinic, workplace | Detailed information is provided in the data collection section of the study. |
| --- | --- | --- | --- |
| 15 | Presence of non-participants | Was there anyone else present apart from the participants and the researchers? | There were no observers. |
| 16 | Description of the sample | What are the important characteristics of the sample, e.g. demographic data, history | Individuals who agreed to participate in the study were included in the study. |

**Data collection**

| 17 | Interview guide | Were questions, prompts, and guidelines provided by the authors? Has it been pilot tested? | Detailed information was given in the Methods section. |
| --- | --- | --- | --- |
| 18 | Repeat interviews | Have there been re-interviews? If yes, how many? | No. |
| 19 | Audio/visual recording | Was audio or visual recording used to collect data in the study? | Interviews were recorded with a voice recorder |
| 20 | Field notes | Were field notes taken during and/or after the interview or focus group? | Responses of all individuals and researcher observations were recorded. |
| 21 | Duration | How long were the interviews or focus groups? | Each interview lasted between 45 and 60 minutes. |
| 22 | Data saturation | Has data saturation been discussed? | Data saturation was discussed. |
| 23 | Transcripts returned | Have transcripts been returned to participants for comments and/or corrections? | No. |

**Area 3: analysis and findings**

| 24 | Number of data coders | How many data coders coded the data? | Two researchers and a third individual coded the data. |
| --- | --- | --- | --- |
| 25 | Description of the coding tree | Did the authors provide a description of the coding tree? | The titles and subtitles in the results section represent the final coding tree. |
| 26 | Derivation of themes | Were the themes predetermined or derived from the data? | Themes were derived from the data. |
| 27 | Software | What software, if any, was used to manage the data? | Data were analyzed manually. |
| 28 | Participant control | Did participants provide feedback on the findings? | No. |

**Reporting**

| 29 | Quotes provided | Are participant quotes presented to illustrate themes/findings? Is each quote identified, e.g. participant number | Conclusion. Participant quotes are presented to illustrate themes/findings. |
| --- | --- | --- | --- |
| 30 | Data and findings consistent | Was there consistency between the data presented and the findings? | Yes |
| 31 | Clarity of main themes | Are the main themes clearly presented in the findings? | Yes |
| 32 | Clarity of small themes | Is there an explanation of the different cases or a discussion of minor issues? | Yes |
